# Identification and characterization of two families of F_420_H_2_-dependent reductases from *Mycobacteria* that catalyse aflatoxin degradation

**DOI:** 10.1111/j.1365-2958.2010.07356.x

**Published:** 2010-11

**Authors:** Matthew C Taylor, Colin J Jackson, David B Tattersall, Nigel French, Thomas S Peat, Janet Newman, Lyndall J Briggs, Gauri V Lapalikar, Peter M Campbell, Colin Scott, Robyn J Russell, John G Oakeshott

**Affiliations:** 1 CSIRO Ecosystem SciencesGPO Box 1700, Canberra, ACT 2601, Australia; 2 Institute de Biologie Structurale41 rue Jules Horowitz, F-38027 Grenoble Cedex 1, France; 3 CSIRO Molecular and Health Technologies343 Royal Parade, Parkville, Vic. 3052, Australia

## Abstract

Aflatoxins are polyaromatic mycotoxins that contaminate a range of food crops as a result of fungal growth and contribute to serious health problems in the developing world because of their toxicity and mutagenicity. Although relatively resistant to biotic degradation, aflatoxins can be metabolized by certain species of *Actinomycetales*. However, the enzymatic basis for their breakdown has not been reported until now. We have identified nine *Mycobacterium smegmatis* enzymes that utilize the deazaflavin cofactor F_420_H_2_ to catalyse the reduction of the α,β-unsaturated ester moiety of aflatoxins, activating the molecules for spontaneous hydrolysis and detoxification. These enzymes belong to two previously uncharacterized F_420_H_2_ dependent reductase (FDR-A and -B) families that are distantly related to the flavin mononucleotide (FMN) dependent pyridoxamine 5′-phosphate oxidases (PNPOxs). We have solved crystal structures of an enzyme from each FDR family and show that they, like the PNPOxs, adopt a split barrel protein fold, although the FDRs also possess an extended and highly charged F_420_H_2_ binding groove. A general role for these enzymes in xenobiotic metabolism is discussed, including the observation that the nitro-reductase Rv3547 from *Mycobacterium tuberculosis* that is responsible for the activation of bicyclic nitroimidazole prodrugs belongs to the FDR-A family.

## Introduction

**Fig. 1:**
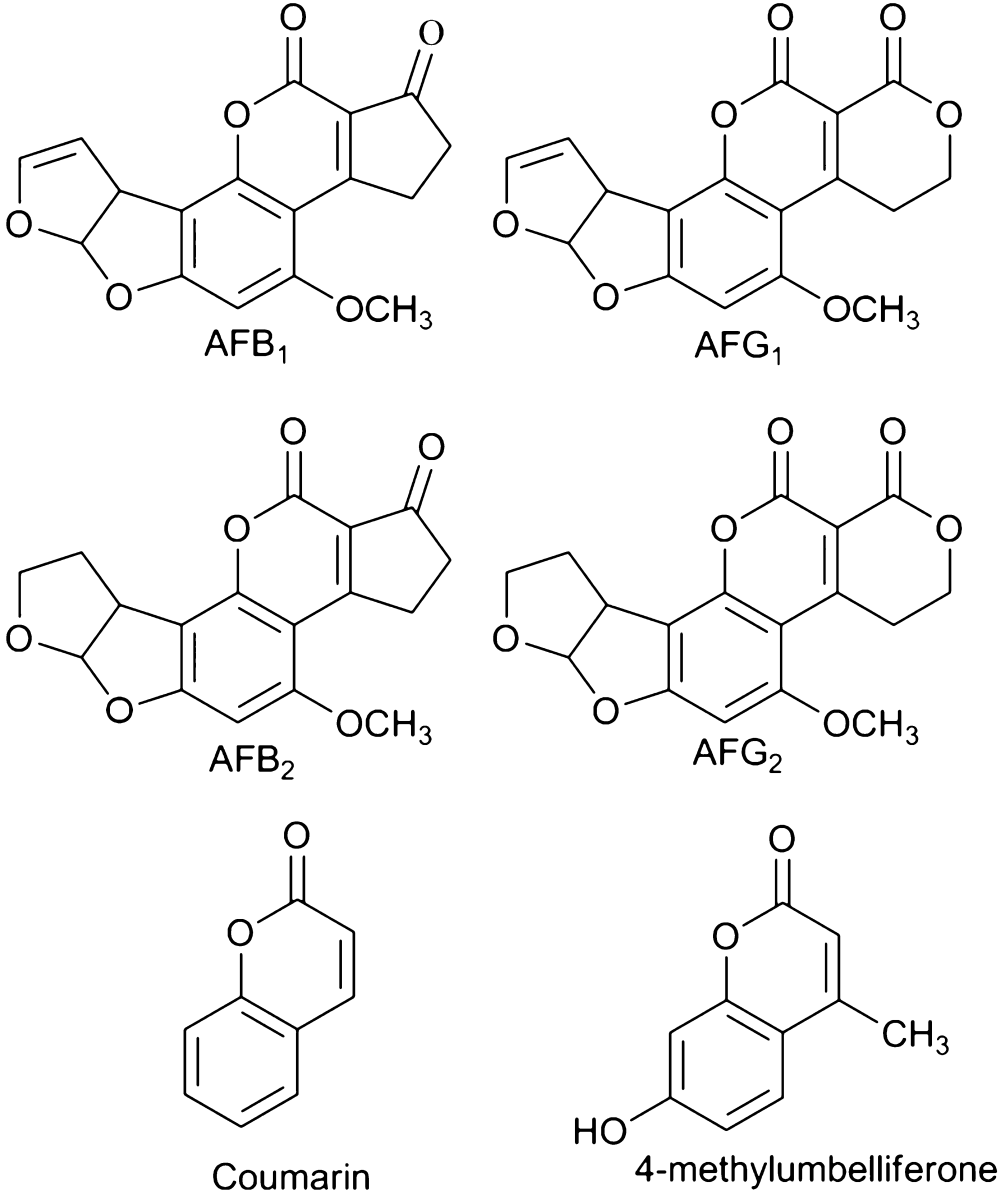
Chemical structures of compounds used in this study.

Aflatoxins are secondary metabolites of various *Aspergillus* species, particularly *A. flavus* and *A. parasiticus*, and are generally separated into two groups: the difurocoumarocyclopentenones (including AFB1 and AFB2) and the difurocoumarolactones (including AFG1 and AFG2) (Fig. 1). Humans can be exposed to aflatoxins via consumption of *Aspergillus-*contaminated maize or nuts, or the milk of animals fed contaminated crops. Recurrent consumption of aflatoxin-contaminated food has been shown to contribute to an increased risk of nutritional deficiencies, immune suppression and hepatocellular carcinoma in several developing nations (Wagacha and Muthomi, 2008). The levels of aflatoxins in internationally traded commodities are tightly regulated by the FAO/WHO and other regulatory bodies (van Egmond *et al*., 2007).

Although higher organisms and many microorganisms have little ability to degrade aflatoxins, they are known to be metabolized by certain fungi and bacteria (Ciegler *et al*., 1966). The bacteria known to degrade aflatoxins are all *Actinomycetales* of the suborder *Corynebacterineae*, such as *Nocardia corynebacterioides* (Ciegler *et al*., 1966; Hormisch *et al*., 2004), *Rhodococcus erythropolis* and *Mycobacterium fluorantheniorans* sp. Nov DSM44556^T^ (Teniola *et al*., 2005). There is evidence that aflatoxin degradation by these species is enzyme catalysed (Smiley and Draughon, 2000; Alberts *et al*., 2006), although the reaction products and the enzymes involved have not previously been described. Because the degradation products of aflatoxins are non-toxic (Ciegler *et al*., 1966; Teniola *et al*., 2005), there is considerable interest in identifying and characterizing the enzymes involved.

Herein, we describe the isolation and characterization of nine F_420_H_2_-dependent reductases (FDRs) from *Mycobacterium smegmatis* that utilize an F_420_H_2_ cofactor to catalyse the reduction of aflatoxins, leading to their spontaneous breakdown. Structural and phylogenetic analysis suggests that these nine enzymes fall into two hitherto unclassified enzyme families that are distantly related to the well characterized FMN-dependent pyridoxamine 5′-phosphate oxidase (PNPOx) family (di Salvo *et al*., 2003). The mechanism of aflatoxin breakdown appears to involve an initial, enzyme-catalysed reduction of the double bond of the α,β-unsaturated ester moiety, followed by spontaneous hydrolysis. The possible cellular functions of these enzymes in xenobiotic metabolism are discussed, as are their potential uses, both as an enzymatic means by which aflatoxins may be detoxified and as potential drug targets, owing to their exclusive presence in *Archea, Actinomycetales* and some species of *γ-Proteobacteria.*

## Results

### Identification of aflatoxin degrading bacterial strains

Seven bacterial strains (*Mycobacterium smegmatis* mc^2^155, *Mycobacterium* sp. ESD, *Arthrobacter* KW-ES, *Arthrobacter* RW thio, *Arthrobacter* RLH#41, *Agrobacterium radiobacter* P230 and *Pseudomonas monteilii* C11) known to degrade various xenobiotics (Sutherland *et al*., 2002; Horne *et al*., 2002a,b) were tested for their ability to degrade aflatoxin AFG1 in liquid culture. Live cultures and cell lysates of the two *Mycobacterium* strains were found to degrade AFG1, while no aflatoxin degradation was observed in live cultures or cell lysates of the other strains tested. The activity of the *Mycobacterium* lysates was lost upon heating, consistent with an enzyme-catalysed reaction, as shown by thin-layer chromatography (TLC; Fig. S1). Although other *Actinomycetales* have previously been shown to degrade aflatoxins (Ciegler *et al*., 1966; Hormisch *et al*., 2004; Teniola *et al*., 2005; Alberts *et al*., 2006), the failure of the *Arthrobacter* cultures tested to degrade AFG1 demonstrates that aflatoxins are not degraded by all *Actinomycetales*. Because the genome of *M. smegmatis* mc^2^155 has been sequenced and annotated, and methodologies for its manipulation in the laboratory are well established, it was used in all subsequent experiments to isolate and identify the gene/enzyme systems responsible for aflatoxin degradation.

### Aflatoxin degradation is cofactor F_420_-dependent

Transposon mutagenesis was used as a first approach to identify the genes/enzymes involved in the degradation of aflatoxin by *M. smegmatis*. After mutagenesis with the EZ::TN <R6Kγori/KAN-2> transposon, 3160 viable isolates were assayed by TLC for their ability to degrade AFG1 over 4 days (Fig. S1). Only five of the 3160 isolates did not degrade AFG1. Sequencing of the region surrounding the transposon insertion site in these colonies showed that four of the insertions disrupted the *fbiC* gene, which catalyses the final step in the biosynthesis of the deazaflavin, Fo, from intermediates of the riboflavin synthesis and tyrosine metabolic pathways (Fig. S2) (Choi *et al*., 2002; Graham *et al*., 2003). Fo is the precursor for the production of the cofactor F_420_ (Graupner and White, 2001). The fifth transposon insertion disrupted the *fgd* gene, which encodes the F_420_-dependent glucose 6-phosphate dehydrogenase (FGD) (Purwantini and Daniels, 1998; Bashiri *et al*., 2008), which is known to recycle F_420_ from its oxidized to its reduced F_420_H_2_ form in *Mycobacteria* (Fig. S2). These data are consistent with an essential role for F_420_ in the degradation of aflatoxins in *M. smegmatis.* Significantly, all the bacterial genera previously shown to degrade aflatoxins (see above) are also known to synthesize F_420_ (Isabelle *et al*., 2002).

To test whether FGD catalysed aflatoxin degradation, it was heterologously expressed in and purified from *Escherichia coli.* As expected, FGD could catalyse the reduction of F_420_ to F_420_H_2_ in the presence of glucose 6-phosphate (Purwantini and Daniels, 1998; Bashiri *et al*., 2008). However, neither FGD in the presence of F_420_/F_420_H_2_, nor F_420_H_2_ in isolation, were able to catalyse aflatoxin breakdown at detectable levels. Thus, although F_420_H_2_ is clearly essential to the reaction, it is most likely involved as a cofactor for another enzyme not identified in this screen.

### Identification and kinetic analysis of two families of F_420_H_2_-dependent aflatoxin degrading enzymes

**Fig. 2:**
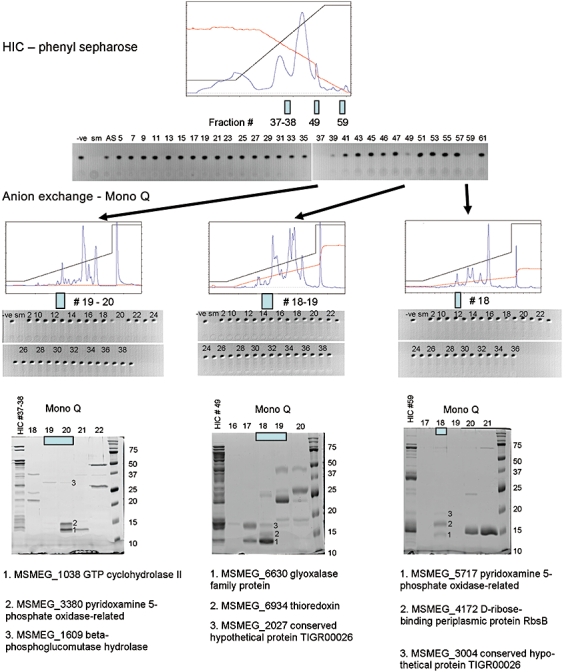
Purification protocol. Protein fractions that showed F_420_H_2_ dependent AFG1 degradation as measured by TLC were separated from *M. smegmatis* extracts. The ammonium sulphate precipitated proteins were first purified by hydrophobic interaction chromatography (HIC). Active fractions were further purified by anion exchange chromatography before separation by SDS-PAGE. Bands were cut from SDS-PAGE gels, digested with trypsin and analysed by LC/MS/MS. Peptides were identified from the annotated *M. smegmatis* genome sequence and corresponding results are shown for some of the excised bands. In the TLCs ‘-ve’ denotes no enzyme negative control and ‘sm’ denotes *M. smegmatis* cell extract positive control.

The enzymes responsible for aflatoxin degradation in *M. smegmatis* were purified from soluble cell extracts. Two protocols were used, one involved ammonium sulphate precipitation, hydrophobic interaction chromatography, anion exchange chromatography and SDS-PAGE (Fig. 2). The second involved ammonium sulphate precipitation, hydrophobic interaction, gel filtration and anion exchange chromatography. Proteins were then identified from active fractions in the final purification steps via tandem mass spectrometry of peptides obtained by tryptic digests and comparison of these fragments to predicted proteins from the *M. smegmatis* genome (Table S1). Only four proteins were identified in both purifications; MSMEG_3380, 3004, 2027 and 5717. The observation that multiple enzymes with the same activity were identified provides an explanation as to why they were not identified by transposon mutagenesis, since they would all need to be simultaneously inactivated to prevent AFG1 degradation.

**Fig. 3:**
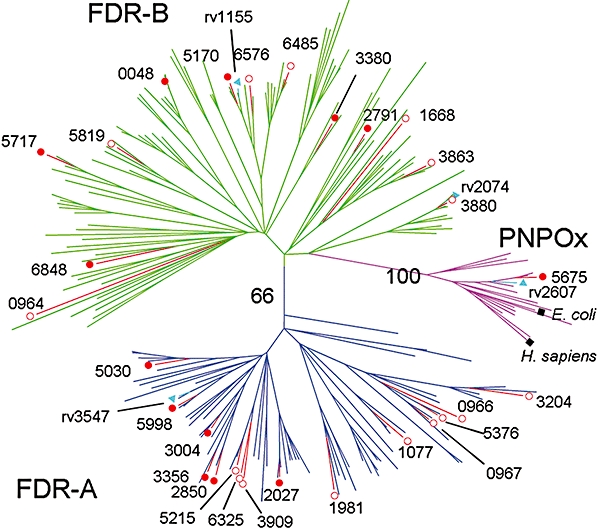
Phylogenetic relationship between the PNPOx, FDR-A and FDR-B families. A condensed tree was constructed from 146 protein sequences of FDRs/PNPOxs from seven species of *Actinomycetales* (*M. smegmatis*, *M. tuberculosis H37Rv*, *M. vanbaalenii*, *Rhodococcus sp. RH1*, *Arthrobacter sp. FB24*, *S. coelicolor*, *Frankia alni* and *Nocardioides* sp. JS614), plus the known PNPOx enzymes from *H. sapiens*, *E. coli*, *Saccharomyces cerevisiae*, *Caenorhabditis elegans* and *Mus musculus*. Solid red circles denote the FDRs described in this paper, and open red circles denote other potential FDRs from *M. smegmatis* (TIGR locus number shown). For clarity, only enzymes from other species that have been previously characterized are labelled: blue triangles denote *M. tuberculosis* enzymes with known structures or previously described functions and black squares denote previously described PNPOxs.

The four proteins identified above were all predicted to be related to the PNPOx family by the protein fold recognition server *PHYRE* (Kelley and Sternberg, 2009), although their relationship is distant (less than 15% amino acid identity). The four proteins have predicted molecular weights, based on their sequences, of approximately 16kDa (Table S1), while proteins in the active *M. smegmatis* fractions were calculated to be approximately 30 kDa by size exclusion chromatography (Fig. S3), suggesting that these four proteins most likely oligomerize as dimers in solution. These results are consistent with the observation that the PNPOx enzymes form homodimers (di Salvo *et al*., 1998). Phylogenetic analysis of 146 PNPOx-like sequences, including 28 present in *M. smegmatis* (Fig. 3 and Fig. S4), shows that the four proteins belong to two distinct families (< 12% amino acid identity), hereafter denoted FDR-A and -B (F_420_H_2_ dependent reductase -A and -B), which are both clearly differentiated from the functionally defined PNPOx family. Although the PNPOx family is widely distributed among both prokaryotes and eukaryotes, the FDR-A and FDR-B families appear to be limited to the *Actinomycetales, Archaea* and some *Proteobacteria,* all of which possess F_420_. Furthermore, the two families are unrelated to other previously characterized F_420_H_2_ dependent reductases, including sulphite reductase (Johnson and Mukhopadhyay, 2005), dinitrophenol reductase (Ebert *et al*., 2001) and the methylenetetrahydromethanopterin reductase complex involved in methanogenesis in *Archaea* (Deppenmeier, 2002; Graham and White, 2002). Interestingly, the only other functionally characterized enzyme that is related to either of these families is the bicyclic nitroimidazole prodrug activating reductase from *M. tuberculosis*, Rv3547 (Manjunatha *et al*., 2006), which has 49% sequence identity to MSMEG_5998 of the FDR-A family (Fig. 3).

The next phase of our work focused on the four *M. smegmatis* enzymes identified by chromatography plus five other members of the FDR-A and FDR-B families in the *M. smegmatis* genome. These five enzymes were identified as close paralogues both by blast analysis and by their retention of a highly conserved loop motif and a putative phosphate binding motif (Fig. S4). The nine genes were cloned, expressed, purified and assayed against the four aflatoxins in the presence of F_420_H_2_ (Table 1). All nine enzymes catalysed measurable degradation of all four aflatoxins tested (AFG1, AFG2, AFB1 and AFB2). AFG1 was found to be the best substrate for all nine enzymes: for example MSMEG_3380 has nearly 50-fold more activity with AFG1 than AFB2. However, the specific activities of the various enzymes differed by more than four orders of magnitude for AFG1. The four enzymes originally identified in the chromatographic experiments were not the most active, perhaps because of purification, expression or stability issues. The three most active enzymes were from the FDR-A family (MSMEG_5998, 2850 and 3356). Kinetic parameters were obtained for MSMEG_5998 with AFB1 (*K*_M_ = 47 ± 6 µM, *K*_cat_ = 63 ± 4 min^−1^); these values could not be obtained for other enzymes because enzyme activity with saturating substrate concentrations could not be measured, owing to the low solubility of the aflatoxins. The fact that all nine enzymes tested had aflatoxin degrading activity suggests that some, if not all, of the other 19 representatives of the two FDR families which we have found by blast analysis of the *M. smegmatis* genome might also catalyse aflatoxin breakdown.

**Table 1:** Specific activities of MSMEG proteins for aflatoxins

		Specific activity (nmol min^−1^ µmol^−1^ enzyme)^a^			
		
Protein family	TIGR Locus^b^	AFG1	AFG2	AFB1	AFB2
FDR-A	5998	83 000 ± 2000	6100 ± 200	9100 ± 700	7700 ± 700
	2850	12 000 ± 2000	8000 ± 500	1300 ± 200	410 ± 60
	3356	8080 ± 70	3100 ± 500	1200 ± 200	1300 ± 200
	3004	1600 ± 100	1260 ± 50	310 ± 60	620 ± 20
	2027	660 ± 40	310 ± 50	220 ± 50	160 ± 20
FDR-B	3380	1600 ± 100	320 ± 40	130 ± 10	33 ± 4
	6848	900 ± 200	430 ± 50	100 ± 10	90 ± 3
	5170	240 ± 40	140 ± 6	330 ± 50	90 ± 2
	5717	3 ± 0.7	3 ± 0.5	3 ± 0.1	2 ± 0.2

a Units are expressed per µmole of the monomer for both FDR-A and -B.

b The TIGR locus number is preceded by ‘MSMEG_’.

Although genome sequences of other *Actinomycete* strains that metabolize aflatoxins, such as *R. erythropolis*, *N. corynebacterioides* and *M. fluoranthenivorans* (Ciegler *et al*., 1966; Hormisch *et al*., 2004; Teniola *et al*., 2005), have not been published, our preliminary screening of several related *Actinomycete* genome sequences (Table S2) shows that each has at least 10 putative FDR-As and FDR-Bs. It thus seems likely that the aflatoxin degrading abilities of the FDRs shown in *M. smegmatis*, and quite likely many other *Actinomycetales*, may originate from FDRs of the A and B families. It is notable that there is only one annotated FDR-B and no annotated FDR-A sequences present in *Arthrobacter* genomes so far published (Table S2), which may explain the lack of aflatoxin degradation observed in the three *Arthrobacter* strains analysed in this study.

### The PNPOx and FDR families are functionally distinct

Because the FDR families have significant sequence similarity to the PNPOxs and the respective cofactors F_420_H_2_ and FMN/FMNH_2_ are structurally related (Fig. S2), we investigated whether there was any cofactor, or catalytic, promiscuity between these enzyme families. Phylogenetic analysis (Fig. 3) identified MSMEG_5675 as the probable *M. smegmatis* PNPOx. In contrast to the expressed FDR-A and -B enzymes, preparations of purified MSMEG_5675 were an intense yellow colour with an absorption maximum at 450 nm, indicative of protein bound to FMN. MSMEG_5675 was confirmed to be a PNPOx by its capacity to catalyse the conversion of pyridoxamine 5′-phosphate (PMP) to pyridoxal 5′-phosphate (PLP) with a specific activity of 0.18 s^−1^, consistent with the catalytic activity of other PNPOxs (di Salvo *et al*., 1998; di Salvo *et al*., 2002). No activity was observed after incubating the FDRs overnight with 100 µM FMN and PMP, suggesting that these enzymes exclusively utilize F_420_H_2_ as a cofactor. Moreover, once FMN was stripped from MSMEG_5675, the apo-enzyme was found to be unable to catalyse aflatoxin degradation in the presence of F_420_H_2_ (data not shown). Thus, our data suggest that the PNPOx and FDR families are functionally distinct and they do not appear to have overlapping activities or cofactor preferences.

### Structures of the FDR-A and FDR-B enzymes

**Fig. 4:**
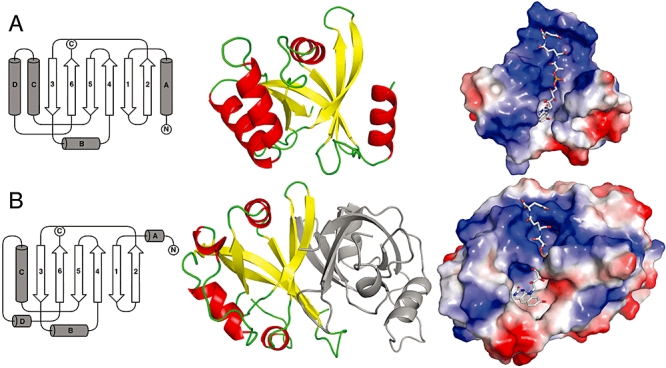
The structures of FDR-A and FDR-B enzymes. A. MSMEG_3356; from left to right, a topology diagram, showing the arrangement of the split barrel of anti-parallel β-sheets, surrounded by three large α-helices at the side and a short α-helix above, a cartoon diagram, and an electrostatic surface potential representation with F_420_H_2_ docked. B. MSMEG_3380; from left to right, a topology diagram of the structure, showing the arrangement of the split barrel of anti-parallel β-sheets, surrounded by one large α-helix at the side and three short α-helices above and below, a cartoon diagram of the 3380 dimer, and an electrostatic surface potential representation with F_420_H_2_ docked.

To gain further insight into the function of these newly identified enzymes, crystal structures of members of the FDR-A (MSMEG_3356) and FDR-B families (MSMEG_3380) were solved to 2.0 and 1.2 Å resolution respectively. MSMEG_3356 and MSMEG_3380 both adopt the PNPOx-like split barrel fold, comprising a central split barrel surrounded by four helices (Fig. 4). MSMEG_3380 exists as a homodimer, while MSMEG_3356 is monomeric.

The structures of MSMEG_3380 and MSMEG_3356 differ significantly, as would be expected given their low sequence similarity; only 88 amino acids could be structurally aligned with an r.m.s.d of 2.2 Å. A DALI (Holm *et al*., 2008) search indicated that the closest known structural homologue to the FDR-A MSMEG_3356 is a putative PNPOx from *Agrobacterium tumefaciens* (3DNH), with just 15% amino acid identity. Thus, it appears that MSMEG_3356 is the first characterized structure of a member of the FDR-A family. The closest relatives of MSMEG_3380 with known structures are the functionally uncharacterized proteins from *M. tuberculosis* Rv1155 and Rv2074 (Biswal *et al*., 2005; 2006; Canaan *et al*., 2005), with amino acid identities of 18% and 24% respectively. These proteins have been putatively classified as PNPOxs, owing to their similarity to the PNPOx family, but no functional analysis has been performed. As shown in our phylogenetic analysis (Fig. 3), they have greater similarity to the FDR-B family than the PNPOxs and we propose that these proteins and MSMEG_3380 comprise the first structures of the FDR-B family.

**Fig. 5:**
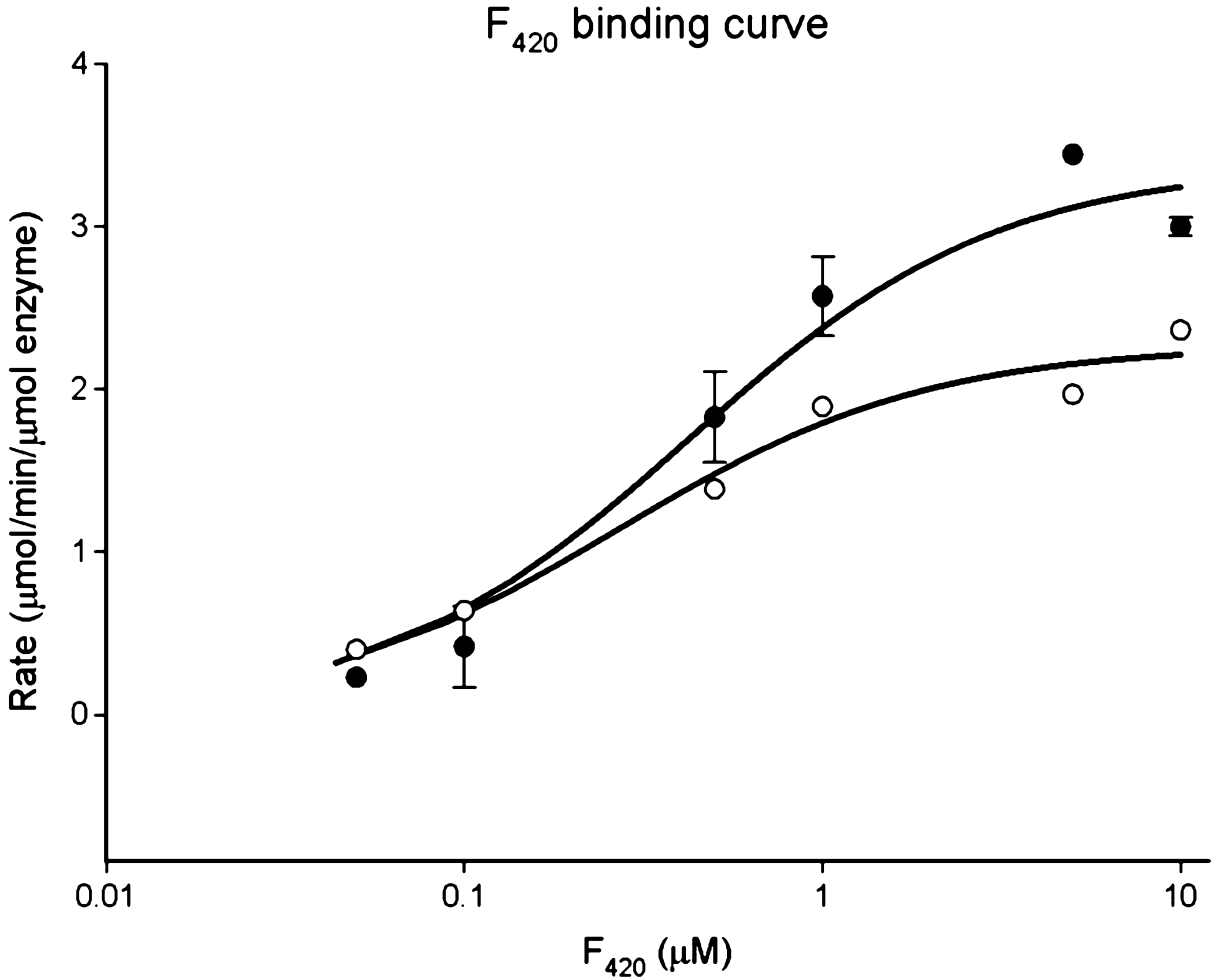
F_420_H_2_ cofactor binding to enzymes 3356 and 3380. The apparent *K*_M_'s for F_420_H_2_ were calculated by measuring the velocity of the reaction with varying concentrations of F_420_H_2_ from 0.05 to 10 µM while maintaining all other components at 100 µM aflatoxin, 1 µM enzyme. For clarity the velocity is expressed as percent of maximum velocity. Empty circles, 3356; filled circles, 3380.

Despite their low similarity, the structures of both the FDRs described here are characterized by large hydrophobic pockets formed by several aromatic residues and a positively charged groove that is principally formed by several lysine and arginine side-chains (Fig. 4). In terms of shape and electrostatic character, these pockets/channels are highly complementary to the F_420_H_2_ cofactor, which possesses an aromatic deazaflavin group and a long, negatively charged, side-chain consisting of a phospho-lactate and a variable number of glutamates. In the absence of a structure of the holoenzyme, we have docked the cofactor, as shown in Fig. 4. Consistent with the level of electrostatic and hydrophobic interactions implied by the docking results, titration curves of activity versus cofactor concentration (Fig. 5) show that both MSMEG_3356 and MSMEG_3380 bind F_420_H_2_ tightly, with apparent *K*_M_ values in the nanomolar range (260 nM and 130 nM, respectively).

**Fig. 6:**
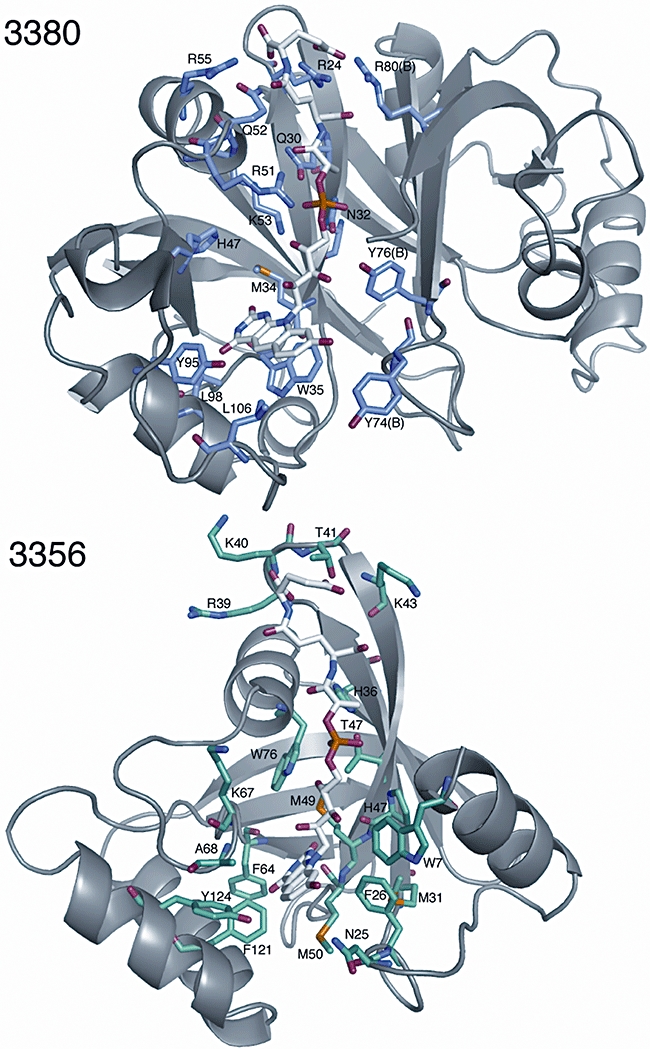
The F_420_H_2_ binding grooves of an FDR-A, MSMEG_3356, and an FDR-B, MSMEG_3380. The deazaflavin pockets are shown to be rich in aromatic and hydrophobic residues, while the side-chain binding grooves are rich in basic residues.

Although the residues forming the putative F_420_H_2_ binding channels and substrate binding pockets occur in homologous regions of the two families, the identities of the residues involved are not conserved (Fig. 6 and Fig. S4). In MSMEG_3356, the deazaflavin/substrate pocket is formed by W7, N25, F26, M31, P48, M49, M50, F64, A68, W76, F121 and Y124, and the positively charged groove is formed by the side-chains of N8, H36, R39, K40, T41, K43, T47 and K67. In MSMEG_3380 the deazaflavin/substrate pocket is formed by M34, W35, Y95, L98 and L106 of monomer A and Y74 and Y76 of monomer B. The positively charged groove is formed by the side-chains of R24, Q30, N32, H47, R51, Q52, K53 and R55 from monomer A and R80 from monomer B. The hydrophobic aflatoxin/deazaflavin binding cavity of MSMEG_3380 is significantly larger than that of MSMEG_3356 (649 Å^3^ versus 411 Å^3^), as measured by the 3V server (Voss and Gerstein, 2010), suggesting that these enzymes are likely to have different substrate ranges.

### The catalytic mechanism of aflatoxin reduction

**Fig. 7:**
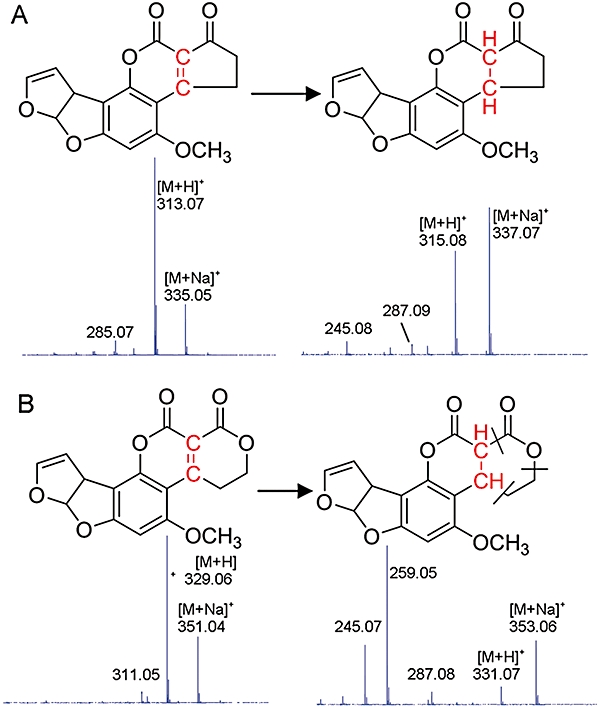
Proposed reaction mechanism of AFB1 and AFG1 reduction by reduced F_420_. A. The proposed reduction mechanism of AFB1, with the mass spectrum of the substrate and product below the chemical structures. B. The mass spectrum and chemical structure of AFG1 and the reduced product.

Liquid chromatography-mass spectrometry (LCMS) analysis of the aflatoxin degradation reaction showed that all of the FDRs tested appeared to catalyse aflatoxin breakdown via the same mechanism for all four substrates, with the ion species of the product differing from the corresponding reactant by 2.02 m/z in each instance (Fig. 7). This is consistent with the reduction of aflatoxin via transfer of two electrons from F_420_H_2_. The reaction product is unstable over time and several low abundance metabolites (possibly generated by spontaneous hydrolysis) appear after an hour. Previously characterized reactions involving AFB1 and AFG1 involved oxidation of the unsaturated carbons of the furan ring by mammalian liver CYP450's (Mishra and Das, 2003), or their spontaneous reduction to form AFB2 and AFG2 (Fig. 1). As the FDRs described here also catalyse reduction of AFB2 and AFG2, it is unlikely that it is the furan moiety that is reduced by these enzymes.

A second candidate moiety for the FDR catalysed reduction is the α,β-unsaturated ester between the lactone rings in AFG1/G2 and the lactone and cyclopentenone rings in AFB1/B2. Indeed, the LCMS-induced fragmentation of AFG1 (Fig. 7) produces ion species (287.08 and 259.05 m/z) that would result from a retro Diels-Alder fragmentation of the lactone ring (loss of CO_2_ and C_2_H_4_ respectively), consistent with destabilization of the ring resulting from reduction of the α,β-unsaturated ester. To test this hypothesis, MSMEG_3380 was incubated with coumarin and 4-methylumbelliferone. Coumarin and 4-methylumbelliferone both contain α,β-unsaturated ester moieties, and lack furan rings. However, the α,β-unsaturated ester moiety in 4-methylumbelliferone is stabilized by a methyl group (Fig. 1). Degradation of coumarin was observed at rates similar to that for aflatoxin (0.5 µmol min^−1^ µmol^−1^), while no degradation of 4-methylumbelliferone was observed. Further experiments will be required to establish details of the catalytic mechanism, but these results suggest that it is the α,β-unsaturated ester moiety that is reduced by the FDRs.

## Discussion

### The possible physiological role of FDRs

Herein we describe two families of enzymes that utilize F_420_H_2_ to degrade aflatoxin. F_420_/F_420_H_2_ is a deazaflavin hydride carrier found in the *Archea, Actinomycetales* and some species of γ-*Proteobacteria.* Although non-essential in *Actinomycetales* in a laboratory setting (Nakano *et al*., 2004), an important role for the cofactor has been documented in a wide variety of metabolic processes. These include methanogenesis (Graham and White, 2002), sulphite reduction (Johnson and Mukhopadhyay, 2005), the degradation of nitroaromatic compounds (Ebert *et al*., 2001; Manjunatha *et al*., 2006; Guerra-Lopez *et al*., 2007; Singh *et al*., 2008), antibiotic resistance (Hasan *et al*., 2010), malachite green decolourization (Guerra-Lopez *et al*., 2007) and the biosynthesis of chlortetracyclines in *Streptomyces* (Nakano *et al*., 2004). It may be that the highly negative redox potential of F_420_H_2_ (Jacobson and Walsh, 1984) is enabling for some of these energetically demanding reactions, including the degradation of aflatoxins by the FDR-A and -B families described here.

It seems unlikely that *M. smegmatis* would need between nine and 28 FDR enzymes specifically to degrade aflatoxin. Indeed, it is questionable as to whether aflatoxins are the primary physiological substrates for any of them; *M. smegmatis* has been found in soil where it may encounter aflatoxins but it is also known to be a human saprophyte where it would be very unlikely to encounter these carcinogens. The *K*_M_ values of these enzymes for aflatoxin were also higher than what might be expected for a physiological substrate; they were above the solubility limit for aflatoxin (approximately 100 µM) for all the enzymes except for the most active aflatoxin degrading FDR that we characterized, MSMEG_5998, which had a *K*_M_ of 47 ± 6 µM for AFB1. By comparison the *K*_M_ value for *E. coli* PNPOx for pyridoxine 5′-phosphate is 2 µM (di Salvo *et al*., 2003). Furthermore, there are significant differences between the active site structure of the FDR-A and -B enzymes, which suggest that they may have a broad substrate range. Our analysis of the substrate binding pockets of MSMEG_3356 and MSMEG_3380 reveal that while the pockets are formed by homologous regions the residues are not conserved. Also, the MSMEG_3380 cavity is significantly larger than that of MSMEG_3356 (649 Å^3^ versus 411 Å^3^). The aflatoxin degrading activity we have characterized may thus reflect inherently broad substrate specificities of the FDR-A and -B families for polyaromatic compounds containing α,β-unsaturated lactones.

Our phylogenetic and genomic analyses (Fig. 3 and Table S2) show that the FDR-A and -B families are widespread among the *Actinomycetales*. Many taxa in this order inhabit environments where aflatoxins are unlikely to be found. However, the FDRs may be involved in other metabolic processes. We can suggest three possibilities. First, we note that other *Actinomycetales*, including some previously found to degrade aflatoxins (Hormisch *et al*., 2004; Teniola *et al*., 2005), have been isolated from environments contaminated with structurally similar polyaromatic hydrocarbons (Willumsen *et al*., 2001; Miller *et al*., 2004). The FDR enzymes may play a role in the degradation of these compounds and hence their utilization as potential carbon and energy sources. Second, some *Actinomycetales* are plant pathogens/symbionts (El-Tarabily and Sivasithamparam, 2006; Schrey and Tarkka, 2008) and many plant antimycobacterials are coumarinyl compounds that are structurally similar to aflatoxins and could be substrates for FDR-A and -B enzymes (Stavri and Gibbons, 2005). Finally, many antibiotic intermediates are potential substrates for these enzymes; for instance the aclacinomycin biosynthetic gene cluster in *Streptomyces galilaeus* contains an uncharacterized gene, *AclJ*, which our phylogenetic analysis suggests is an FDR-A enzyme (Chung *et al*., 2002). Some FDR-A and -B enzymes may therefore be involved in antibiotic biosynthesis.

### Potential industrial and medical applications

The FDR-catalysed aflatoxin breakdown described in this work constitutes significant detoxification of these compounds, as previous studies have shown that removal of the lactone ring of AFB1 reduces its mutagenicity by 450-fold and its acute toxicity by 18-fold (Lee *et al*., 1981). Furthermore, pre-incubation with various *Actinomycetales* has been demonstrated to reduce the mutagenicity and toxicity of aflatoxin to animals (Ciegler *et al*., 1966; Alberts *et al*., 2006; Tejada-Castaneda *et al*., 2008). Accordingly, there is a potential use for these enzymes in decontaminating aflatoxin-contaminated food crops, either as a transgenic input trait for the crop or in a post-harvest application. There are precedents for the effective decontamination of two other mycotoxins (zearalenone and deoxynivalenol) in maize and rice by transgenic expression of the corresponding bacterial detoxifying enzymes (Igawa *et al*., 2007; Ohsato *et al*., 2007). Coexpression of the genes required for the biosynthesis of reduced F_420_ would also be required for the FDRs to be effective as an input trait and there are now many examples of plants being engineered with multiple genes (Taverniers *et al*., 2008). The identification of non-pathogenic strains of *Actinomycetales* that have the capacity to degrade aflatoxin may provide a less technologically intensive biocontrol approach. Indeed, inoculation of *Aspergillus* growth media with *Streptomyces* species has been shown to protect peanuts from aflatoxin contamination in the laboratory (Zucchi *et al*., 2008).

The exclusivity of these FDRs to certain *Actinobacteria* is an important ‘point of difference’ that can be targeted in the development of drugs, particularly considering the presence of several human pathogens within this phylum, including *M. tuberculosis*. For instance, the candidate anti-tuberculosis drug PA-824 is now known to be a prodrug that is metabolized to its active form through the activity of an FDR-A (Rv3547) (Manjunatha *et al*., 2006). Our identification and structural characterization of the first members of these FDR families thus raises the possibility of rational design of improved prodrugs with even greater activity and specificity. However, before detailed analysis of the catalytic mechanism is possible, structures of the holoenzymes (F_420_H_2_ : Enz) and, ideally, a ternary complex will be required, since substantial conformational changes are often seen in cofactor and substrate binding in related enzymes, such as PNPOx (Safo *et al*., 2005).

### Comparison between the FDRs and PNPOxs

Structurally and phylogenetically, the FDRs and PNPOxs appear to have a common evolutionary origin, yet these enzyme families have evolved to utilize different cofactors. Notwithstanding their structural similarities there are at least two important chemical differences between the two cofactors. One is the lower redox midpoint potential of F_420_ in solvent at pH 7.5 (−350 mV for F_420_ versus −230 mV for FMN) (Draper and Ingraham, 1968; Jacobson and Walsh, 1984). The other is that F_420_ is not stable as a semiquinone and may only catalyse a two electron reduction, while FMN may catalyse one or two electron reduction and oxidation reactions (Goldberg *et al*., 1981). Indeed, owing to this, the chemistry that is generally catalysed by the two enzyme families is opposite (oxidation by the PNPOxs versus reduction by the FDRs). We suggest it is this difference in catalytic activity that has resulted in the evolutionary expansion of the FDRs in *M. smegmatis* (28 genes) compared with the single PNPOx gene; the reducing power of F_420_H_2_ may have allowed these enzymes to catalyse the reduction of a wide variety of compounds, particularly xenobiotics, and provided a selective pressure for duplication and specialization.

There are also significant differences in the mechanisms of cofactor recycling between the FDRs and the PNPOxs. PNPOxs bind FMN with high affinity, *K*_D_ 30 nM (Wada and Snell, 1961), and require molecular oxygen as the electron acceptor (Pogell, 1958; Notheis *et al*., 1995). In contrast, there is a requirement for F_420_ to be enzymatically reduced by FGD, at least in *Mycobacteria* and *Nocardia* (Purwantini *et al*., 1997). It is currently impossible to determine whether a common evolutionary progenitor of the FDRs and PNPOxs would have used either FMN, F_420_H_2_, or perhaps a different molecule again (such as a common phosphorylated intermediate from the biosynthetic pathways of FMN and F_420_; Fig. S2) as a cofactor, but these families of enzymes have the potential to advance our understanding of how enzymes evolve to utilize the catalytic power that different cofactors provide.

## Experimental procedures

### Bacterial strains, chemicals

Aflatoxins B_1_, B_2_, G_1_ and G_2_ were obtained from Sigma-Aldrich and Fermentek (Israel). All bacterial strains were previously isolated in our laboratory (Sutherland *et al*., 2002; Horne *et al*., 2002a,b), except for *M. smegmatis* mc^2^155, which was obtained from Dr H. Billman-Jacob (University of Melbourne, Australia). F_420_ was purified from *M. smegmatis* soluble fractions according to previously published methods (Isabelle *et al*., 2002).

### Identification of aflatoxin degrading bacteria

Bacterial strains were first grown on Luria–Bertani (LB) agar before inoculation into Peptone Yeast extract Broth (PYB; 9 g/l peptone, 4.5 g l^−1^ yeast extract, 23 mM Na_2_HPO_4_, 88 mM KH_2_PO_4_, 9 mM NaCl, pH 6.0) supplemented with 4 µg ml^−1^ AFG1 or 6 µg ml^−1^ AFB1, and incubation at 28°C for 48 h on an orbital shaker (200 rpm). Five microlitres of each reaction mixture was spotted and dried onto silica gel 60 *F*_254_ TLC plates (Merck, Germany). Chloroform/acetone/acetic acid (40:10:1 by volume) was used as the developing solvent and aflatoxin fluorescence was detected by viewing under ultraviolet light (365 nm). Images of TLC plates were recorded by an AlphaImager 2200 Imaging System (Alpha Innotech, USA) fitted with an ethidium bromide bandpass filter.

### Transposon mutagenesis of *M. smegmatis*

Random insertion mutants of *M. smegmatis* mc^2^155 were generated with the EZ::TN <R6Kγori/KAN-2> insertion kit (Epicentre, USA). The EZ::TN <R6Kγori/KAN-2> tnp transposase complex (1 µl) was used to electroporate 100 µl of electrocompetent *M. smegmatis* mc^2^155 cells, which was then plated onto LB agar plates containing 20 µg ml^−1^ kanamycin. Approximately 2000 mutants were obtained per transformation. Transposon insertion mutants were individually inoculated into 2 ml square wells of 96-deep-well growth blocks (Axygen, USA) containing 200 µl of PYB supplemented with 20 µg ml^−1^ kanamycin and 4 µg ml^−1^ aflatoxin G_1_. The growth blocks were sealed with silicone mats (Axygen) and incubated for 3 days (37°C at 200 rpm) before 5 µl of each culture was examined for aflatoxin degradation by TLC. Mutants that exhibited detectable growth but had reduced ability to degrade AFG1 compared with wild-type cells were selected for further analysis. The genomic regions of selected mutants containing the EZ::TN <R6Kγori/KAN-2> transposon were isolated by plasmid rescue. Genomic DNA was isolated using the Bactozol DNA isolation kit (Molecular Research Center, USA), digested with EcoRI, self-ligated and used to electroporate *E. coli* TransforMax EC100D pir-116 (Epicentre, USA). Transformants containing the transposon-interrupted *M. smegmatis* DNA were selected by plating onto LB agar containing 40 µg ml^−1^ kanamycin. The resulting plasmids were isolated and the genomic regions flanking the transposon were sequenced using primers supplied with the EZ::TN <R6Kγori/KAN-2> insertion kit.

### Identification of AFG1-degrading proteins in *M. smegmatis*

Protein fractions with AFG1 activity were purified from *M. smegmatis* by ammonium sulphate precipitation, hydrophobic interaction chromatography, anion exchange chromatography and gel filtration chromatography, as detailed below. Proteins from active fractions were visualized by SDS-PAGE and Coomasie staining. Mass spectrometry of tryptic digests with an Agilent XCT ion trap mass spectrometer (Campbell *et al*., 2008) was used to identify the proteins from protein bands excised from SDS-PAGE and active fractions from gel filtration chromatography. Agilent's Spectrum Mill software was used to match the data with annotated protein sequences in the *M. smegmatis* mc^2^155 CMR database (Peterson *et al*., 2001).

### Chromatography of *M. smegmatis* proteins

*Mycobacterium smegmatis* was grown for 4 days before centrifugation at 10 000 *g* and lysis via sonication with glass beads in 20 mM Tris-HCl, pH 7.5, with 1 mg ml^−1^ lysozyme, 5 mM DTT and 1 mM PMSF. Saturated (NH_4_)_2_SO_4_ solution (0°C) was added to the soluble fraction to a concentration of 40% before centrifugation at 20 000 *g*. A second (NH_4_)_2_SO_4_ cut (70%) of the soluble fraction was made and the resulting pellet was resuspended in 1 M (NH_4_)_2_SO_4_, 20 mM Tris-HCl, pH 7.5, and clarified via filtration with a 0.22 µm filter. A Biologic HR FPLC system (Bio-Rad, USA) was used during the subsequent chromatographic separation steps. Hydrophobic interaction chromatography (HIC) was performed using a 200 ml phenyl sepharose high performance column (GE Healthcare, USA) equilibrated with 1 M (NH_4_)_2_SO_4_, 20 mM Tris-HCl, pH 7.5. After the resolubilized (NH_4_)_2_SO_4_ pellet was loaded onto the column, proteins were eluted over a linear gradient [1 M–0 M (NH_4_)_2_SO_4_] over 100 ml. Active fractions were pooled and dialysed against 20 mM Tris-HCl, pH 7.5 prior to anion exchange chromatography with a MonoQ HR 5/5 column (GE Healthcare), equilibrated with 20 mM Tris-HCl, pH 7.5. The dialysed fractions were loaded onto the column and eluted over a linear gradient (0–0.5 M NaCl) over 20 column volumes. For size exclusion chromatography, as used in the second purification, HIC active fractions were pooled and loaded onto a Superdex 200 Hi Load 26/60 gel filtration column (GE Healthcare), equilibrated with 150 mM NaCl, 20 mM Tris-HCl, pH 7.5. Gel filtration chromatography standards (GE Healthcare) were run in parallel to allow estimation of the size of the proteins in solution.

### Phylogeny

Homologues of the four aflatoxin degrading enzymes identified by reverse genetics were identified by searching the TIGR CMR peptide and NCBI protein databases, using the blast and CDD (Marchler-Bauer *et al*., 2007). MEGA4 (Tamura *et al*., 2007) was used for the phylogenetic analysis of a limited data set of 146 amino acid sequences from seven different *Actinomycetales* (*M. smegmatis*, *M. tuberculosis* H37Rv, *M. vanbaalenii*, *Rhodococcus* sp. RH1, *Arthrobacter* sp. FB24, *S. coelicolor*, *Frankia alni* and *Nocardioides* sp. JS614), plus the known PNPOx enzymes from *H. sapiens*, *E. coli*, *Saccharomyces cerevisiae*, *Caenorhabditis elegans* and *Mus musculus*. Protein sequences were aligned by the Gonnett algorithm and the tree constructed from the N-terminal sequences using the neighbour-joining method with pairwise deletion, poisson correction and 1000 bootstrap replicates. The 28 *M. smegmatis* PNPOx-like sequences are referred to by their TIGR locus numbers (MSMEG_).

### Cloning, expression and purification of recombinant proteins

Genes were amplified from *M. smegmatis* mc^2^155 genomic DNA using Platinum high fidelity *Taq* polymerase (Invitrogen, USA) using the primer pairs in Table S3. The amplicons were recombined into the Gateway expression vector, pDEST17 (Invitrogen, USA) and the sequences confirmed by capillary electrophoresis (Micromon, Australia). Proteins were expressed in *E. coli* BL21-AI (Invitrogen) and purified by nickel agarose affinity chromatography using a 1 ml Ni-NTA superflow column (Qiagen, Germany). Proteins were stored at 4°C in 50 mM Tris-HCl pH 7.5. The FDR MSMEG_5998, which was purified as exclusion bodies, was refolded following the methods of Whitbread *et al*. (2005). Protein concentrations were determined by measuring absorbance at 280 nm using a NanoDrop Spectrophotometer ND1000 and calculated based on the extinction co-efficient for each protein determined using Vector NTI (Invitrogen). FMN was stripped from MSMEG_5675 by dialysis, as described previously (di Salvo *et al*., 1998), and this was confirmed by loss of the FMN absorption maxima at 450 nm. For protein crystallography, MSMEG_3380 and MSMEG_3356 were cloned into pDEST17, with a TEV-cleavage site designed into the forward primer (Table S3). Expression and purification were performed as described previously (Jackson *et al*., 2008). The *fgd* gene was cloned into the NdeI and BamHI sites of pET14b (Novagen, Germany) and expressed in Tuner cells (Novagen) by induction with 0.4 mM Isopropyl-β-D-thiogalactopyranoside (IPTG).

### Enzyme assays

The FGD activity was estimated using the previously described spectrophotometric assay for F_420_ to F_420_H_2_ reduction (Purwantini and Daniels, 1996). One unit of FGD activity was defined as the amount of enzyme required to reduce one µmole of F_420_ per min. Assays of aflatoxin degradation activity were conducted in either 10 µl or 20 µl reaction volumes, in the dark, at room temperature, using reaction mix (30 µM aflatoxin, 10 µM F_420_, 0.2 U µl^−1^ FGD, 2.5 mM glucose 6-phosphate, 20 mM Tris-HCl, pH 7.5) and enzyme. Reactions were incubated for 0.5 min to 24 h depending on the enzyme and aflatoxin used. The reaction was stopped by the addition of formic acid to a final concentration of 2% and incubated on ice for 15 min. Protein was pelleted by centrifugation for 5 min at 14 000 *g*. Samples were injected onto an Agilent Zorbax XDB C18 column (3.5 µm, 2.1 × 30 mm). Aflatoxin concentrations were quantified using an Agilent 1200 series binary HPLC running isocratically with 30% methanol and 0.5% acetic acid and detected at 365 nm and quantified using Chemstation software (Agilent). Results are given in specific activity units because of the low aqueous solubility of aflatoxin [∼100 µM for AFB1 (Grant and Phillips, 1998)], which prevented the estimation of kinetic parameters.

### LC/MS assays

The reaction products of aflatoxin reduction were determined using an Agilent 1100 Series Binary LC with diode array detector and in-line Time of Flight Mass Spectrometer (MSD TOF). Samples were separated on an Agilent Zorbax XDB C18 column (3.5 µm, 2.1 × 30 mm) over a gradient of 5% acetonitrile (v/v) and 0.1% formic acid (v/v) from 0.5 min to 20% acetonitrile at 2 min, and then increased to 50% acetonitrile at 10 min, at a flow rate of 0.3 ml min^−1^. Reaction products were analysed using Analyst QS software.

### Structural analysis

The crystallization and data collection of 3380 have been previously reported (Jackson *et al*., 2008). The crystallization of Seleno-Methionine 3356 was performed in 2 µl hanging drops with 20 mg ml^−1^ enzyme and a reservoir solution of 36% PEG-MME 5000, 0.1 M sodium acetate, pH 5.5. Seleno-Methionine labelled crystals were soaked in cryoprotectant [35% PEG 10K, 10 mM Tris-HCl, pH 8.5 for MSMEG_3380 and reservoir solution with 10% glycerol and 10% ethylene glycol (v/v) for MSMEG_3356] before vitrification in liquid nitrogen. Both data sets were collected at the Australian Synchrotron, 3380 on the PX1 beamline and 3356 on the PX2 microfocus beamline. The use of the microfocus beamline for 3356 was necessitated by severe non-merohedral twinning of the crystals; crystals were scanned for regions displaying a minimum of twinning. Intensity data were collected at 100 K using a wavelength corresponding to the SeMet edge at 0.9793 Å and a 1.0° oscillation angle per image. Diffraction data were integrated and scaled using the programs *MOSFLM* (Leslie, 2006) and *SCALA* (Evans, 2006). Details of the data collection are given in Table 2. Based on systematic absences, the 3380 and 3356 crystals were assigned to the P2_1_2_1_2_1_ and P2_1_ space groups respectively. The SHELX suite of programs (Schneider and Sheldrick, 2002) was used for experimental phasing. Four and eight selenium sites were found in the asymmetric units of 3380 and 3356, respectively, using SHELXD, and were used to calculate phases. After refinement and density modification, a significant amount of both structures, corresponding to a dimer in the asymmetric unit of MSMEG_3380 and a tetramer in the asymmetric unit of MSMEG_3358, could be auto-built using ARP/wARP (Perrakis *et al*., 2001), yielding a preliminary *R*_free_ values of 0.304 and 0.421 for 3380 and 3356 respectively. Further maximum-likelihood refinement for 3380, or twin-refinement with a twin fraction of 7% for 3356, using REFMAC5 (Murshudov *et al*., 1997), interspersed with manual building in COOT (Emsley and Cowtan, 2004), addition of water molecules and final anisotropic (for 3380) refinement bought the final *R*_work_/*R*_free_ values to 16.8/19.1 and 19.0/25.5 for 3380 and 3356 respectively. The F_420_ cofactor of the FDRs was docked into the structures using the docking program CDOCKER (Wu *et al*., 2003), as implemented in the Accelrys Discovery Studio. Hydrogens were added to cofactor and enzyme and the CHARMm forcefield was used. Slight manual adjustment of the top hit was made using COOT. Analysis of cavity volumes was performed by submitting the coordinates to the 3V server (http://3vee.molmovdb.org) (Voss and Gerstein, 2010), and all images were created using Pymol (http://www.pymol.org).

**Table 2:** Data collection and refinement statistics for the crystallography

Crystal	MSMEG_3356	MSMEG_3380

PDB ID	3H96	3F7E
Space group	P1211	P212121
Unit cell (Å)	a = 59.65, b = 71.15, c = 63.84	a = 56.86, b = 65.35, c = 69.74
Unit cell (°)	α = 90, β = 90.39, γ = 90	α = 90, β = 90, γ = 90
Data collection		
Resolution (Å)^a^	45.7–2.0 (2.11–2.00)	22–1.23 (1.23–1.30)
Unique reflections	34129	73957
Redundancy	7.2 (6.4)	6.4 (6.2)
<I/σ(I)>	5.7 (3.3)	6.8 (1.5)
Completeness (%)	94.5 (91.5)	97.6 (95.4)
*R*_sym_ (%)^b^	10.5 (17.4)	7.2 (49.4)
Refinement		
No. reflections work/free	31 389/2743	70 232/3725
Resolution range	45.7–2.0 (2.05–2.00)	20–1.23 (1.26–1.23)
*R*_work_/*R*_free_ (%)^c^	19.0/25.5 (20.9/27.7)	16.8/19.1 (20.0/23.1)
R.m.s deviations		
Lengths (Å)	0.025	0.012
Angles (°)	1.84	1.59

a Values in parenthesis are for the highest resolution shell.

b *R*_symm_ = |*I_j_* – *I_j_*|/*I_j_*, where I_j_ is the averaged intensity for symmetry related reflections.

c *R*_work_ = |*F*_(obs)_ – *F*_(calc)_|/*F*_(obs)_; 5% of the data that were excluded from the refinement were used to calculate *R*_free_.
