# Supplementary material for: Identification and characterization of two families of F420H2-dependent reductases from Mycobacteria that catalyse aflatoxin degradation
Source: Mol Microbiol. 2010 Nov;78(3):561–75. doi: 10.1111/j.1365-2958.2010.07356.x (PMC3034190; doi:10.1111/j.1365-2958.2010.07356.x)
Supplement: Supplementary file 1 [file mmi0078-0561-SD1.pdf]

Supplementary Methods–

PNPOx PMP to PLP conversion assay. The rate of PMP conversion to PLP by the FMN dependent MSMEG_5675 was determined using 0.5 µM of the enzyme incubated with 200µM PMP and 100µM FMN in Tris pH 7.5, time points were taken every time 10 minutes and quantified on an Agilent 1200 HPLC based on previously published methods (Bisp *et al.*, 2002). Briefly, PLP and PMP were separated on a Phenomenex (USA) Synergi 2.5µ hydro-RP 100A column equilibrated with 1-octanesulfonic acid and 1.2mM triethylamine with 33mM phosphoric acid, adjusted to pH 2.15 with KOH (mobile phase A) at 1ml/min. The compounds were separated over a linear gradient from 0% acetonitrile to 20% acetonitrile over 1 min followed by an increase to 40% acetonitrile over 4.5 minutes, the column was subsequently re-equilibrated in mobile phase A, prior to the subsequent injection. Rates were determined as the loss of the substrate against a standard curve and calculated using Chemstation.

Fig. S1 Thin layer chromatograms of aflatoxin degradation by *M. smegmatis* and transposon mutated strains. For panel A, *M. smegmatis* soluble extract was incubated with aflatoxin overnight and separated by TLC: lane 1, aflatoxin only; lane 2, aflatoxin and soluble extract; lane 3 aflatoxin and boiled soluble extract. Panel B is a representative TLC of cultures of *M. smegmatis* transposon mutants incubated for 19 hours with aflatoxin; the culture names are shown. Sequencing of the three mutants unable to degrade aflatoxin shown here revealed that the transposon had inserted in the N-terminal half of FGD for 8.11G and the C-terminal half of FbiC for 17.7G and 19.7E.

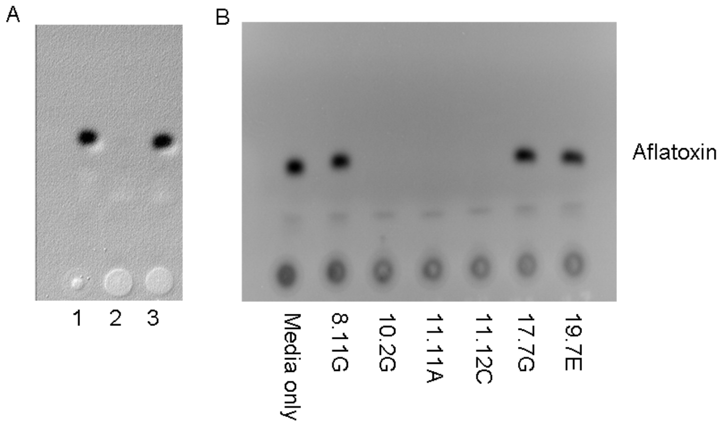

Fig. S2. Biosynthetic pathways of the cofactors F_{420} and FMN. Both FMN (yellow box) and F_{420} (green box) utilize the same biosynthetic pathway from GTP to a phosphorylated intermediate (5-amino-6-ribityl-2butanone 4-phosphate). Riboflavin is synthesized by the condensation of the dephosphorylated intermediate with a four-carbon precursor (red) derived from ribulose 5-phosphate and subsequently re-phosphorylated to form FMN. Alternatively, in *Mycobacteria*, FbiC catalyzes the formation of Fo by condensation of the dephosphorylated intermediate with the 4-hydroxy-phenylpyruvate (red). FbiA and FbiB catalyze the addition of the phospholactone and γ-linked glutamates, respectively. FGD utilizes glucose-6-phosphate to reduce F_{420} to $F_{420}H_2$, which is subsequently utilized as a cofactor for FDR catalyzed reactions.

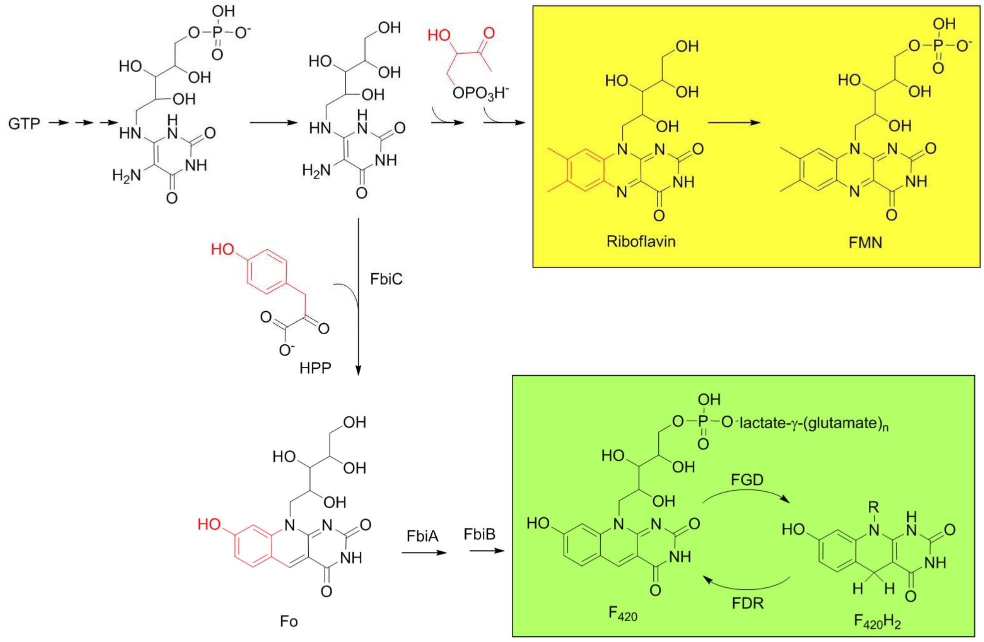

Fig. S3. Gel filtration chromatography of AFG1 degrading fractions from *M. smegmatis*. Molecular weight standards (panels A and E) and three fractions from *M. smegmatis* (panels B - D) were separated by gel filtration chromatography. The retention time and molecular weight of the standards are shown, and the active fractions from the *M. smegmatis* purified fractions are highlighted. The molecular weights of the active fractions were calculated by plotting the molecular weight standards. The three *M. smegmatis* fractions were purified by a 40-70% ammonium sulfate cut followed by hydrophobic interaction chromatography prior to the gel filtration chromatography.

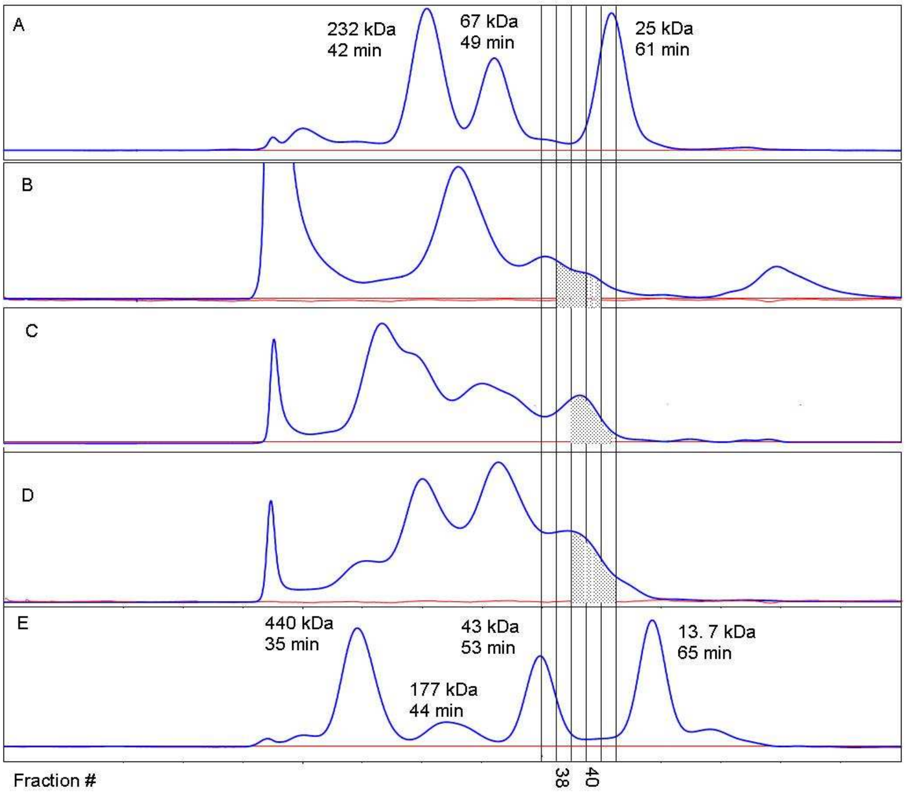

Fig. S4. Sequence alignment and structural domains. Amino acid sequences of the FDR-A and -B enzymes from *M. smegmatis* were aligned and the secondary structural elements of MSMEG_3356 and MSMEG_3380 shown (panel A and B, respectively). Panel C shows the alignment of the PNPOx enzymes from *M. smegmatis* (MSMEG_5675), *M. tuberculosis* (rv2607), *H. sapiens* and *E. coli* with the secondary structural elements of rv2607 shown (Pedelacq *et al.*, 2006). β strands are shown as blue arrows and α helices as red cylinders. Identical amino acids are shown as red letters, conserved residues as blue, homologous residues as green, and weakly similar grey. The yellow triangles highlight the highly conserved glycine residue in the loop between β strands 1 and 2. The yellow diamond highlights the conserved (putative) phosphate binding residue, Trp in FDR-A and Lys in FDR-B and PNPOx.

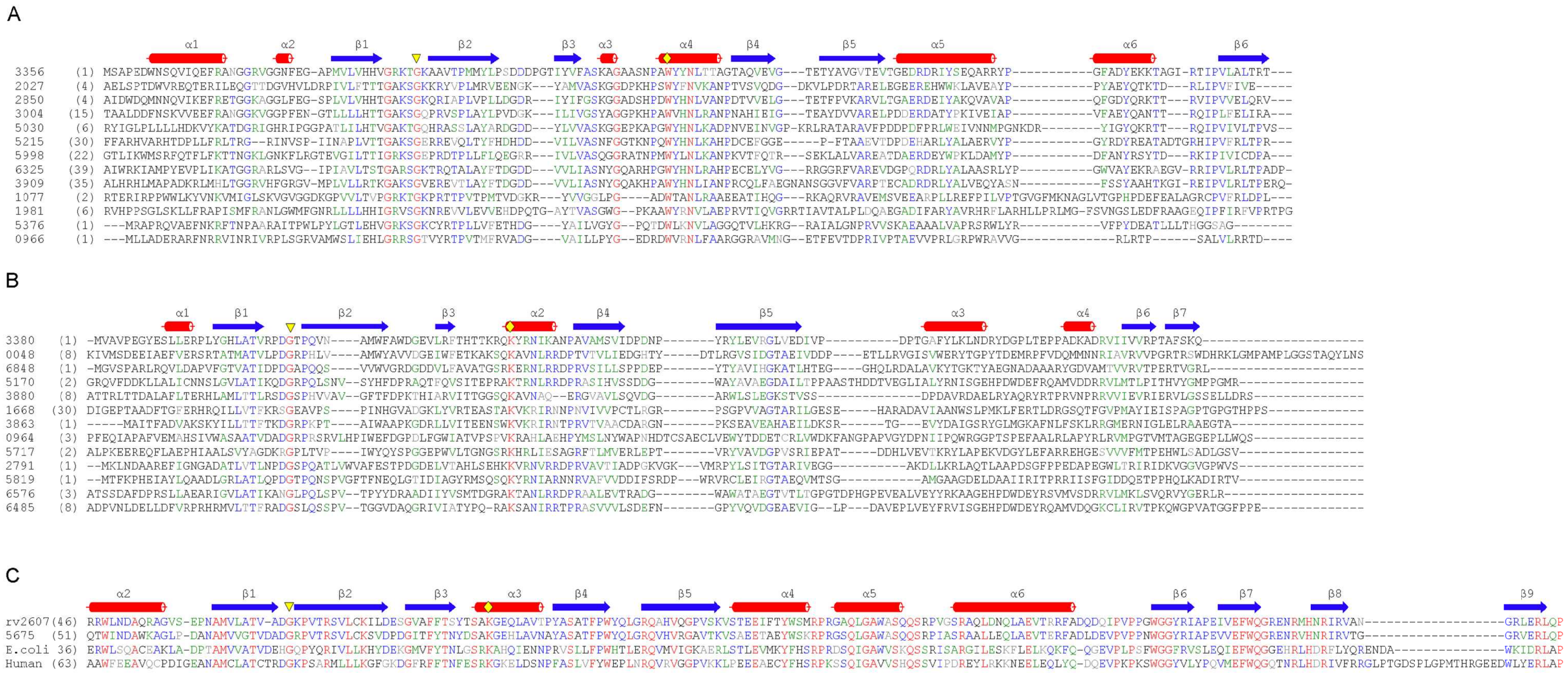
A
α1 α2 β1 β2 β3 α3 α4 β4 β5 α5 α6 β6
3356 (1)
2027 (4)
2850 (4)
3004 (15)
5030 (6)
5215 (30)
5998 (22)
6325 (39)
3909 (35)
1077 (2)
1981 (6)
5376 (1)
0966 (1)
B
α1 β1 β2 β3 α2 β4 β5 α3 α4 β6 β7
3380 (1)
0048 (8)
6848 (1)
5170 (2)
3880 (8)
1668 (30)
3863 (1)
0964 (3)
5717 (2)
2791 (1)
5819 (1)
6576 (3)
6485 (8)
C
α2 β1 β2 β3 α3 β4 β5 α4 α5 α6 β6 β7 β8 β9
rv2607(46)
5675 (51)
E.coli 36)
Human (63)

Table S1 – Proteomics/mass spec data

TIGR locus tag MSMEG	NCBI accession number	MW	Distinct peptides	%AA coverage	Mean Peptide Spectral Intensity	Pfam designation	E value	Phyre Fold E value to Rv2991*
3380	ABK72884	14629	4	38	1.42 x10^{6}	pfam01243	6x10^{-13}	2.5x10^{-14}
2027	ABK75334	18021	5	55	2.42 x10^{7}	pfam04075	4x10^{-33}	6.3x10^{-3}
5717	ABK72164	15873	3	24	6.49 x10^{6}	pfam01243	7x10^{-6}	6.6x10^{-15}
3004	ABK74167	16489	6	56	6.55 x10^{6}	pfam04075	8x10^{-32}	1.3x10^{-3}

*Phyre analysis of all proteins gave the lowest E value scores to *M. tuberculosis* protein rv2991 (non published sequence coordinates available on Phyre). They belong to the FMN-binding split barrel superfamily, PNP-oxidase like enzymes.

Table S2 – Number of putative FDR-A and -B enzymes in the *Actinomycetale* species referred to in this study.

**Species**	**Genome accession number**	**# of putative proteins**		
		**PNPOx**	**FDR-A**	**FDR-B**
M. smegmatis	CP000480	1	15	13
M. tuberculosis H37Rv	BX842578	1	6	7
M. vanbaalenii	CP000511	1	13	20
Rhodococcus jostii sp. RH1[1]	CP000631	1	13	12
Arthobacter sp FB24[2]	CP000454	1	0	1
Streptomyces coelicolor	AL939116	1	4	11
Frankia alni	CT573213	1	18	17
Nocardioides sp. JS614	CP000509	1	4	5
M. Gilvum[3]	CP000656	1	12	11

1. *Rhodococcus jostii sp. RH1* shares 96% nucleotide identity in 16S DNA sequence to the aflatoxin degrading *N. corynebacterioides* (DSM20151) referred to in the text. No 16S DNA was available for *R. erythropolis* (DSM 14303).
2. The genome sequence of *Arthobacter sp FB24* was used for *Arthrobacter sp. KW-ES* as it shared 98% sequence identity to the 16S DNA.
3. *M. Gilvum* shared 97% identity to the 16S DNA sequence of the aflatoxin degrading *M. fluoranthenivorans* (DSM 44556)

Table S3 – Primers for the expression work

Primer name	Family	Sequence
5126 attB1	FbiC	AAAAAGCAGGCTTAGTGGATGAATCTCGACTC
5126 attB2	FbiC	AGAAAGCTGGGTACTACGCGGCCAGGGGCGC
3380 attB1	FDR-P	AAAAAGCAGGCTCCATGGTCGCCGTGC
3380 attB2	FDR-P	AGAAAGCTGGGTCTACTGCTTGCTGAACG
5717 attB1	FDR-P	AAAAAGCAGGCTCGATGGCCCTTCCCAAAG
5717 attB2	FDR-P	AGAAAGCTGGGTTCAGACCGAGCCCAGG
0048 attB1	FDR-P	AAAAAGCAGGCTTAATGTCCGACGAGGAGATC
0048 attB2	FDR-P	AGAAAGCTGGGTATCACGAGTTCAGGTACTG
2791 attB1	FDR-P	AAAAAGCAGGCTTAATGAAACTCAACGACGCCG
2791 attB2	FDR-P	AGAAAGCTGGGTATCACGACACCCAGGGGCC
5675 attB1	FDR-P	AAAAAGCAGGCTTAGTGGGGATACCGGACGAT
5675 attB2	FDR-P	AGAAAGCTGGGTACTAGGGCTGGAGCCGTTC
5819 attB1	FDR-P	AAAAAGCAGGCTTATTGAGGTCCTACCGTGGC
5819 attB2	FDR-P	AGAAAGCTGGGTATCAGACCGTTCGTATATC
6848 attB1	FDR-P	AAAAAGCAGGCTTAGTGGGGACGTTTGTCATTTC
6848 attB2	FDR-P	AGAAAGCTGGGTATCAGAGCCGGCCGACGGT
5170 attB1	FDR-P	AAAAAGCAGGCTTAATGGGGGCGCGTCAGGTG
5170 attB2	FDR-P	AGAAAGCTGGGTATCAGCGCATGCCGGGCGGCA
2027 attB1	FDR-A	AAAAAGCAGGCTTAGTGACACCTGCGCAC
2027 attB2	FDR-A	AGAAAGCTGGGTCTATTCGACGATGAACACG
3004 attB1	FDR-A	AAAAAGCAGGCTTAATGACCGACGATTCGATC
3004 attB2	FDR-A	AGAAAGCTGGGTGGCGCGGATCAATTCG
3356 attB1	FDR-A	AAAAAGCAGGCTTAATGAGCGCACCTGAGGAC
3356 attB2	FDR-A	AGAAAGCTGGGTACTACGTGCGCGTGAGGGC
5998 attB1	FDR-A	AAAAAGCAGGCTTAATGGCCGACACTTCCCGT
5998 attB2	FDR-A	AGAAAGCTGGGTACTAAGCCGGGTCGCAGAT
2850 attB1	FDR-A	AAAAAGCAGGCTTAATGAACAACCAGGTGATC
2850 attB2	FDR-A	AGAAAGCTGGGTATCAGACGCGCTGCAACTC
5030 attB1	FDR-A	AAAAAGCAGGCTTATTGCTGCACGACAAGGTC
5030 attB2	FDR-A	AGAAAGCTGGGTACTAGCTCACGGGCGTCAG
AttB1 adapter		GGGGACAAGTTTGTACAAAAAAGCAGGCT
AttB2 adapter		GGGGACCACTTTGTACAAGAAAGCTGGGT
FGD forward	FGD	CGCATATGGCTGAATTGAAGCTAGGTTAC
FGD reverse	FGD	CGGGATCCTCAGGCCAGCTTGCGCAACCG
pDONR201 seq forward		TCGCGTTAACGCTAGCATGGATCTC
pDONR201 seq reverse		GTAACATCAGAGATTTTGAGACAC
3380TEV attB1		**GGGGACAAGTTTGTACAAAAAAGCAGGCT**TAGAAAACCTGTATTTTCAGGGA*ATG*GTCGCCGTGCCCGA
3356TEV attB1		**GGGGACAAGTTTGTACAAAAAAGCAGGCT**TAGAAAACCTGTATTTT

		CAGGGA
